# Manganese as a Potential Confounder of Serum Prolactin

**DOI:** 10.1289/ehp.114-a458a

**Published:** 2006-08

**Authors:** Michael Aschner

**Affiliations:** Department of Pediatrics, Vanderbilt University Medical Center, Nashville, Tennessee, E-mail: michael.aschner@vanderbilt.edu

de Burbure et al. (2006) elegantly demonstrated that dopaminergic markers in the serum, namely prolactin and homovanillic acid, are affected in children exposed to cadmium, lead, mercury, and arsenic. These findings, at low environmental exposure levels, reinforce the potential of these metals to perturb dopaminergic function and optimal development.

In spite of the strengths of the article, de Burbure et al. (2006) overlooked an important potential confounder. Specifically, the authors should consider the possibility that manganese confounded their data; if so, the data set should be reexamined. A strong relationship between manganese exposure and serum prolactin levels has been raised in multiple studies. Although prolactin levels serve as a direct measurement of monoamines or their metabolites in peripheral tissues (e.g., blood platelets, plasma, urine), plasma prolactin is also an indirect indicator of dopaminergic functioning, a target for excessive exposure to manganese (Mutti and Smargiassi 1998; Smargiassi and Mutti 1999). A concordance between neurocognitive deficits and manganese exposure also exists, including a recent study in children exposed to water manganese concentrations exceeding 300 μg/L (Wasserman et al. 2006). A significant and positive correlation between blood manganese concentrations and prolactin levels in cord blood has also been established (Tasker et al. 2004). Other examples abound, although negative relationships between manganese and prolactin have also been reported (Roels et al. 1992).

The potential that exposure to manganese contributed to or confounded the effects of the four metals on serum prolactin levels in the cohorts studied by de Burbue et al. (2006) should be considered. If samples are available for additional analysis, correlations between manganese exposure and prolactin would be beneficial and welcomed by various health forums as the debate on safe manganese exposure levels and sensitive health effect biomarkers continues.
